# *N*-(3,4-Diethoxy­phen­yl)acetamide

**DOI:** 10.1107/S1600536809018042

**Published:** 2009-05-20

**Authors:** Pei-Hua Ma, Kai-Zhi Zhou, Mei-Lian Sun, Xiu-Mei Zhao, Xin Xiao

**Affiliations:** a Key Laboratory of Macrocyclic and Supramolecular Chemistry of Guizhou Province, Guizhou University, Guiyang 550025, People’s Republic of China

## Abstract

In the title compound, C_12_H_17_NO_3_, the conformations of the N—H and C=O bonds are *anti* to each other. In the crystal structure, N—H⋯O hydrogen-bond inter­actions help to establish the packing.

## Related literature

For the use of acetamides in the synthesis of biologically active compounds, see: Koike *et al.* (1999 ▶). The benzanilide core is present in compounds with a wide range of biological activity and benzanilides and benzamides are also used extensively in organic synthesis (Saeed *et al.*, 2008 ▶). Various *N*-substituted benzamides exhibit potent anti­emetic activity, see: Vega-Noverola *et al.* (1989 ▶).
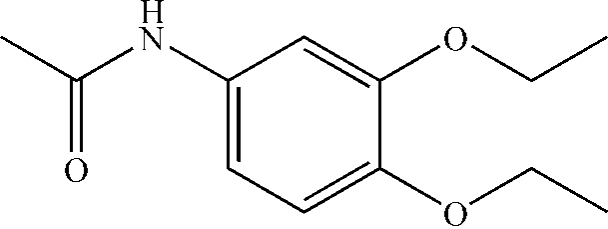


## Experimental

### 

#### Crystal data

C_12_H_17_NO_3_
*M* *_r_* = 223.27
Monoclinic, 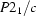

*a* = 15.563 (8) Å
*b* = 8.661 (6) Å
*c* = 9.305 (7) Å
β = 101.773 (14)°
*V* = 1227.8 (14) Å^3^
*Z* = 4
Mo *K*α radiation
μ = 0.09 mm^−1^
*T* = 293 K
0.24 × 0.21 × 0.20 mm

#### Data collection

Bruker SMART CCD area-detector diffractometer
Absorption correction: multi-scan (*SADABS*; Bruker, 2005 ▶) *T*_min_ = 0.971, *T*_max_ = 0.975
6295 measured reflections
2155 independent reflections
1570 reflections with *I* > 2σ(*I*)
*R*_int_ = 0.034

#### Refinement

*R*[*F* ^2^ > 2σ(*F* ^2^)] = 0.044
*wR*(*F* ^2^) = 0.119
*S* = 1.08
2155 reflections
145 parameters
H-atom parameters constrained
Δρ_max_ = 0.16 e Å^−3^
Δρ_min_ = −0.25 e Å^−3^

### 

Data collection: *SMART* (Bruker, 2002 ▶); cell refinement: *SAINT* (Bruker, 2002 ▶); data reduction: *SAINT*; program(s) used to solve structure: *SHELXS97* (Sheldrick, 2008 ▶); program(s) used to refine structure: *SHELXL97* (Sheldrick, 2008 ▶); molecular graphics: *ORTEP-3 for Windows* (Farrugia, 1997 ▶); software used to prepare material for publication: *WinGX* (Farrugia, 1999 ▶).

## Supplementary Material

Crystal structure: contains datablocks global, I. DOI: 10.1107/S1600536809018042/at2786sup1.cif

Structure factors: contains datablocks I. DOI: 10.1107/S1600536809018042/at2786Isup2.hkl

Additional supplementary materials:  crystallographic information; 3D view; checkCIF report

## Figures and Tables

**Table 1:** Hydrogen-bond geometry (Å, °)

*D*—H⋯*A*	*D*—H	H⋯*A*	*D*⋯*A*	*D*—H⋯*A*
N1—H1⋯O3^i^	0.86	2.08	2.915 (2)	164

Symmetry code: (i) 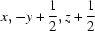.

**Fig. 1.**
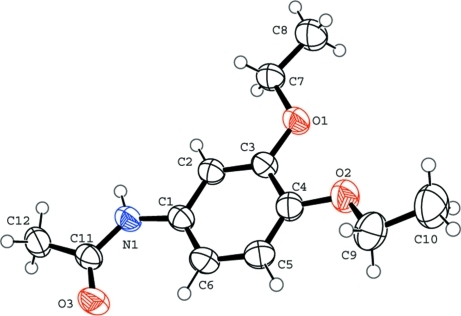
The molecular structure of (I) showing the atom-labelling scheme. Displacement ellipsoids are drawn at the 50% probability level.

## supplementary crystallographic information

### Comment

Acetamide is an important class of medical intermidate. Many biologically active compounds are synthesized by using acetamide (Koike *et al.*, 1999). The benzanilide core is present in compounds with a wide range of biological activity and benzanilides and benzamides are also used extensively in organic synthesis (Saeed *et al.*, 2008). Various N-substituted benzamides exhibit potent antiemetic activity (Vega-Noverola *et al.*, 1989). The crystal structure determination of the title compound (I) has been carried out in order to elucidate the molecular conformation.

The molecule of the title compound, (Fig. 1), consists of a phenylacetamide group and two ethoxyl groups. The conformations of the N—H and C=O bonds are anti to each other. The C10—C9—O2—C4 and C8—C7—O1—C3 torsion angles are -173.61 (15)° and 178.46 (15)°, respectively. The title compound forms intermolecular H bonds whereas the N1 act as hydrogen-bond donor and the O3 act as hydrogen-bond acceptor, the distance of the N1—H1···O3 hydrogen bond is 2.915 (2) Å (Table 1). In the crystal structure, N—H···O hydrogen bonds interactions may help to establish the packing.

### Experimental

Ferrous powder (2.20 g, 0.039 mol), water (15 ml) and acetic acid (3 ml) were reflux for 4 h, the reaction mixture was cooled to room temperature. Then a solution of 1,2-diethoxy-4-nitrobenzene (2.10 g, 0.01 mol) in acetic acid (50 ml) was added to the mixture, the solution was reflux for 6 h. the mixture was filtered, and the resulting solution was added to water (150 ml), much white precipitate was appeared, the mixture was filtered again, the solid product was dissolved in 80 ml ethanol. and then set aside for five days to obtain colourless crystals [yield: 53%].

### Refinement

All other H atoms were placed in calculated positions and refined as riding, with C—H = 0.93–0.97 Å, N—H = 0.86 Å, and *U*_iso_(H) = 1.2–1.5 *U*_eq_(C,*N*).

### Crystal data

**Table:**

C_12_H_17_NO_3_	*F*(000) = 480
*M**_r_* = 223.27	*D*_x_ = 1.208 Mg m^−^^3^
Monoclinic, *P*2_1_/*c*	Mo *K*α radiation, λ = 0.71073 Å
Hall symbol: -P 2ybc	Cell parameters from 2155 reflections
*a* = 15.563 (8) Å	θ = 1.3–25.0°
*b* = 8.661 (6) Å	µ = 0.09 mm^−^^1^
*c* = 9.305 (7) Å	*T* = 293 K
β = 101.773 (14)°	Block, colourless
*V* = 1227.8 (14) Å^3^	0.24 × 0.21 × 0.20 mm
*Z* = 4	

### Data collection

**Table:**

Bruker SMART CCD area-detector diffractometer	2155 independent reflections
Radiation source: fine-focus sealed tube	1570 reflections with *I* > 2σ(*I*)
graphite	*R*_int_ = 0.034
φ and ω scans	θ_max_ = 25.0°, θ_min_ = 1.3°
Absorption correction: multi-scan (*SADABS*; Bruker, 2005)	*h* = −18→16
*T*_min_ = 0.971, *T*_max_ = 0.975	*k* = −10→10
6295 measured reflections	*l* = −10→11

### Refinement

**Table:**

Refinement on *F*^2^	Primary atom site location: structure-invariant direct methods
Least-squares matrix: full	Secondary atom site location: difference Fourier map
*R*[*F*^2^ > 2σ(*F*^2^)] = 0.044	Hydrogen site location: inferred from neighbouring sites
*wR*(*F*^2^) = 0.119	H-atom parameters constrained
*S* = 1.08	*w* = 1/[σ^2^(*F*_o_^2^) + (0.0639*P*)^2^] where *P* = (*F*_o_^2^ + 2*F*_c_^2^)/3
2155 reflections	(Δ/σ)_max_ < 0.001
145 parameters	Δρ_max_ = 0.16 e Å^−^^3^
0 restraints	Δρ_min_ = −0.25 e Å^−^^3^

### Special details

**Table:**

Geometry. All e.s.d.'s (except the e.s.d. in the dihedral angle between two l.s. planes) are estimated using the full covariance matrix. The cell e.s.d.'s are taken into account individually in the estimation of e.s.d.'s in distances, angles and torsion angles; correlations between e.s.d.'s in cell parameters are only used when they are defined by crystal symmetry. An approximate (isotropic) treatment of cell e.s.d.'s is used for estimating e.s.d.'s involving l.s. planes.
Refinement. Refinement of *F*^2^ against ALL reflections. The weighted *R*-factor *wR* and goodness of fit *S* are based on *F*^2^, conventional *R*-factors *R* are based on *F*, with *F* set to zero for negative *F*^2^. The threshold expression of *F*^2^ > σ(*F*^2^) is used only for calculating *R*-factors(gt) *etc*. and is not relevant to the choice of reflections for refinement. *R*-factors based on *F*^2^ are statistically about twice as large as those based on *F*, and *R*- factors based on ALL data will be even larger.

### Fractional atomic coordinates and isotropic or equivalent isotropic displacement parameters (Å^2^)

**Table:**

	*x*	*y*	*z*	*U*_iso_*/*U*_eq_	
C1	0.36147 (10)	0.08455 (17)	0.48242 (16)	0.0426 (4)	
C2	0.32424 (10)	0.07894 (18)	0.60656 (15)	0.0451 (4)	
H2	0.3519	0.1289	0.6920	0.054*	
C3	0.24680 (10)	0.00019 (18)	0.60451 (16)	0.0445 (4)	
C4	0.20432 (11)	−0.07482 (19)	0.47506 (17)	0.0479 (4)	
C5	0.24235 (11)	−0.0712 (2)	0.35439 (18)	0.0542 (5)	
H5	0.2153	−0.1223	0.2693	0.065*	
C6	0.32090 (11)	0.00790 (19)	0.35673 (17)	0.0513 (4)	
H6	0.3458	0.0089	0.2739	0.062*	
C7	0.24108 (11)	0.0806 (2)	0.84850 (17)	0.0554 (5)	
H7A	0.2403	0.1891	0.8225	0.067*	
H7B	0.3013	0.0508	0.8887	0.067*	
C8	0.18566 (14)	0.0537 (3)	0.9582 (2)	0.0759 (6)	
H8A	0.2081	0.1128	1.0451	0.114*	
H8B	0.1866	−0.0541	0.9827	0.114*	
H8C	0.1265	0.0850	0.9179	0.114*	
C9	0.06962 (12)	−0.1888 (2)	0.3449 (2)	0.0656 (5)	
H9A	0.0967	−0.2673	0.2944	0.079*	
H9B	0.0573	−0.0991	0.2817	0.079*	
C10	−0.01353 (12)	−0.2493 (3)	0.3827 (3)	0.0869 (7)	
H10A	−0.0539	−0.2776	0.2942	0.130*	
H10B	−0.0394	−0.1707	0.4330	0.130*	
H10C	−0.0004	−0.3382	0.4449	0.130*	
C11	0.48850 (10)	0.20423 (18)	0.39666 (17)	0.0446 (4)	
C12	0.56454 (11)	0.3114 (2)	0.44698 (19)	0.0569 (5)	
H12A	0.5671	0.3393	0.5476	0.085*	
H12B	0.5570	0.4026	0.3872	0.085*	
H12C	0.6181	0.2606	0.4383	0.085*	
N1	0.43955 (8)	0.17335 (14)	0.49706 (14)	0.0459 (4)	
H1	0.4583	0.2131	0.5824	0.055*	
O1	0.20629 (7)	−0.01068 (13)	0.72098 (11)	0.0559 (4)	
O2	0.12688 (7)	−0.14790 (14)	0.48136 (13)	0.0626 (4)	
O3	0.47167 (8)	0.15207 (13)	0.27060 (12)	0.0587 (4)	

### Atomic displacement parameters (Å^2^)

**Table:**

	*U*^11^	*U*^22^	*U*^33^	*U*^12^	*U*^13^	*U*^23^
C1	0.0448 (9)	0.0439 (9)	0.0407 (8)	0.0034 (7)	0.0126 (7)	0.0045 (7)
C2	0.0490 (10)	0.0488 (9)	0.0385 (9)	−0.0023 (7)	0.0114 (7)	−0.0007 (7)
C3	0.0481 (10)	0.0458 (9)	0.0427 (9)	−0.0007 (7)	0.0162 (7)	0.0006 (7)
C4	0.0476 (10)	0.0486 (10)	0.0475 (9)	−0.0042 (8)	0.0103 (7)	−0.0004 (7)
C5	0.0615 (11)	0.0593 (11)	0.0419 (9)	−0.0073 (9)	0.0105 (8)	−0.0074 (8)
C6	0.0602 (11)	0.0569 (10)	0.0398 (9)	−0.0008 (8)	0.0174 (8)	−0.0008 (8)
C7	0.0614 (11)	0.0640 (11)	0.0437 (9)	−0.0105 (9)	0.0172 (8)	−0.0088 (8)
C8	0.0879 (15)	0.0933 (15)	0.0524 (11)	−0.0225 (12)	0.0279 (10)	−0.0150 (10)
C9	0.0592 (12)	0.0656 (12)	0.0658 (12)	−0.0083 (9)	−0.0018 (9)	−0.0002 (9)
C10	0.0583 (13)	0.0939 (17)	0.1049 (17)	−0.0174 (12)	0.0083 (12)	−0.0065 (13)
C11	0.0517 (10)	0.0432 (9)	0.0422 (9)	0.0096 (7)	0.0169 (7)	0.0094 (7)
C12	0.0590 (11)	0.0557 (10)	0.0611 (11)	−0.0030 (8)	0.0239 (9)	0.0089 (8)
N1	0.0501 (8)	0.0527 (8)	0.0375 (7)	−0.0036 (6)	0.0151 (6)	0.0004 (6)
O1	0.0606 (8)	0.0681 (8)	0.0443 (6)	−0.0172 (6)	0.0226 (6)	−0.0093 (6)
O2	0.0585 (8)	0.0752 (9)	0.0550 (7)	−0.0218 (6)	0.0133 (6)	−0.0094 (6)
O3	0.0723 (8)	0.0664 (8)	0.0423 (7)	0.0002 (6)	0.0231 (6)	0.0029 (5)

### Geometric parameters (Å, °)

**Table:**

C1—C6	1.380 (2)	C8—H8B	0.9600
C1—C2	1.395 (2)	C8—H8C	0.9600
C1—N1	1.421 (2)	C9—O2	1.439 (2)
C2—C3	1.382 (2)	C9—C10	1.503 (3)
C2—H2	0.9300	C9—H9A	0.9700
C3—O1	1.3636 (19)	C9—H9B	0.9700
C3—C4	1.409 (2)	C10—H10A	0.9600
C4—C5	1.372 (2)	C10—H10B	0.9600
C4—O2	1.3732 (19)	C10—H10C	0.9600
C5—C6	1.398 (2)	C11—O3	1.2342 (19)
C5—H5	0.9300	C11—N1	1.3471 (19)
C6—H6	0.9300	C11—C12	1.502 (2)
C7—O1	1.437 (2)	C12—H12A	0.9600
C7—C8	1.483 (2)	C12—H12B	0.9600
C7—H7A	0.9700	C12—H12C	0.9600
C7—H7B	0.9700	N1—H1	0.8600
C8—H8A	0.9600		
			
C6—C1—C2	119.30 (15)	H8A—C8—H8C	109.5
C6—C1—N1	125.16 (14)	H8B—C8—H8C	109.5
C2—C1—N1	115.54 (13)	O2—C9—C10	106.70 (16)
C3—C2—C1	120.95 (14)	O2—C9—H9A	110.4
C3—C2—H2	119.5	C10—C9—H9A	110.4
C1—C2—H2	119.5	O2—C9—H9B	110.4
O1—C3—C2	124.51 (14)	C10—C9—H9B	110.4
O1—C3—C4	115.80 (14)	H9A—C9—H9B	108.6
C2—C3—C4	119.69 (14)	C9—C10—H10A	109.5
C5—C4—O2	125.06 (15)	C9—C10—H10B	109.5
C5—C4—C3	118.91 (15)	H10A—C10—H10B	109.5
O2—C4—C3	116.03 (14)	C9—C10—H10C	109.5
C4—C5—C6	121.37 (15)	H10A—C10—H10C	109.5
C4—C5—H5	119.3	H10B—C10—H10C	109.5
C6—C5—H5	119.3	O3—C11—N1	123.10 (16)
C1—C6—C5	119.75 (15)	O3—C11—C12	121.56 (15)
C1—C6—H6	120.1	N1—C11—C12	115.32 (14)
C5—C6—H6	120.1	C11—C12—H12A	109.5
O1—C7—C8	107.94 (14)	C11—C12—H12B	109.5
O1—C7—H7A	110.1	H12A—C12—H12B	109.5
C8—C7—H7A	110.1	C11—C12—H12C	109.5
O1—C7—H7B	110.1	H12A—C12—H12C	109.5
C8—C7—H7B	110.1	H12B—C12—H12C	109.5
H7A—C7—H7B	108.4	C11—N1—C1	129.38 (14)
C7—C8—H8A	109.5	C11—N1—H1	115.3
C7—C8—H8B	109.5	C1—N1—H1	115.3
H8A—C8—H8B	109.5	C3—O1—C7	117.45 (13)
C7—C8—H8C	109.5	C4—O2—C9	117.83 (13)
			
C6—C1—C2—C3	−1.1 (2)	C4—C5—C6—C1	−0.2 (3)
N1—C1—C2—C3	178.02 (13)	O3—C11—N1—C1	−2.1 (2)
C1—C2—C3—O1	−179.99 (14)	C12—C11—N1—C1	176.25 (14)
C1—C2—C3—C4	−0.4 (2)	C6—C1—N1—C11	1.4 (2)
O1—C3—C4—C5	−178.74 (14)	C2—C1—N1—C11	−177.71 (14)
C2—C3—C4—C5	1.7 (2)	C2—C3—O1—C7	7.5 (2)
O1—C3—C4—O2	0.8 (2)	C4—C3—O1—C7	−172.04 (14)
C2—C3—C4—O2	−178.79 (14)	C8—C7—O1—C3	178.46 (15)
O2—C4—C5—C6	179.12 (15)	C5—C4—O2—C9	−17.2 (2)
C3—C4—C5—C6	−1.4 (3)	C3—C4—O2—C9	163.33 (15)
C2—C1—C6—C5	1.4 (2)	C10—C9—O2—C4	−173.61 (15)
N1—C1—C6—C5	−177.64 (15)		

### Hydrogen-bond geometry (Å, °)

**Table:**

*D*—H···*A*	*D*—H	H···*A*	*D*···*A*	*D*—H···*A*
N1—H1···O3^i^	0.86	2.08	2.915 (2)	164

Symmetry codes: (i) *x*, −*y*+1/2, *z*+1/2.
